# Investigation on microbial inactivation and urea decomposition in human urine during thermal storage

**DOI:** 10.2166/washdev.2017.142

**Published:** 2017-06-05

**Authors:** Xiaoqin Zhou, Yajie Li, Zifu Li, Yue Xi, Sayed Mohammad Nazim Uddin, Yang Zhang

**Affiliations:** 1 **Xiaoqin Zhou Yajie Li Zifu Li** (corresponding author)**Yang Zhang** School of Energy and Environmental Engineering, Beijing Key Laboratory of Resource-oriented Treatment of Industrial Pollutants, University of Science and Technology Beijing, Beijing 100083, China; 2 **Yue Xi** College of Environmental Sciences and Engineering, Peking University, Beijing, China; 3 **Sayed Mohammad Nazim Uddin** Department of Geography, University of Victoria, Victoria, BC, Canada; 4 **Yang Zhang** China-ASEAN Environmental Cooperation Center, Ministry of Environmental Protection, Beijing, China

**Keywords:** human urine, hygienization, stabilization, thermal treatment

## Abstract

The World Health Organization suggests storing human urine for at least 6 months at 20°C prior to application as fertilizer to reduce the potential health risks from pathogenic organisms. Such a storage condition for human urine,however,not only requires along period of time and large space but also ignores the risk of nitrogen losses. In this study, human urine underwent thermal treatment during storage to improve disinfection and to inhibit urea hydrolysis. Microbial indicators such as *Escherichia coli* and fecal coliforms and the concentration of ammonia/ammonium were investigated in urine samples that were stored at 60°C and 70°C. Both the inactivation of indicators and decomposition of urea improved under storage temperatures of 60°C and 70°C compared with storage under ambient temperature. Therefore, human urine is recommended to be stored at 70°C for 7 days for hygienic and stabilization purposes. Under this storage condition, pH is maintained below 8.0 and ammonia/ammonium content is maintained at approximately 800 mg/L.

## INTRODUCTION

The concept of source-separated human feces and urine collection to promote sustainable sanitation solutions from the local to the global level has gained increased attention in recent decades (Boh *et al*. 2013; O'Neal & Boyer 2013). Given that urine contains available nutrients for plant growth, including nitro-gen, phosphorus, and potassium (Lind *et al*. 2001; Karak & Bhattacharyya 2011), urine is a good source of green (Akpan-Idiok *et al*. 2012) and multinutrient fertilizer (Karak & Bhattacharyya 20n), as well as providing a range of environmental benefits (Tidaker *et al*. 2007). Several technologies on the

This is an Open Access article distributed under the terms of the Creative Commons Attribution Licence (CC BY 4.0), which permits copying, adaptation and redistribution, provided the original work is properly cited (http://creativecommons.org/licenses/by/4.0/). doi: 10.2166/washdev.2017.142

laboratory or industriai level have been utilized to recover nutrients from urine for agricultural purposes (Pronk & Koné 2009). These technologies include struvite recovery via precipitation or electricity generation (Sakthivel *et al*. 2012; You *et al*. 2014), nitrification and distillation (Udert & Wachter 2012), and stripping and absorption (Basakçilardan-Kabakci *et al*. 2007; Zhang *et al*. 2015). The direct application of urine as fertilizer after proper storage is favored in rural and suburban areas.

Urea is biochemically hydrolyzed into ammonium $\left({\text{NH}}_{\text{4}}^{\text{+}}\right)$, bicarbonate $\left({\text{HCO}}_{\text{3}}^{\text{-}}\right)$, and hydroxyl (OH^-^), thus increasing pH and ammonium concentration (Vinnerâs *et al* 2006; Jons-son & Vinnerâs 2007) and exerting lethal effects on microorganisms. Therefore, pH, storage duration, and temperature are the major factors that influence the storage process of human urine. Moreover, temperature is a crucial parameter that influences microbial inactivation rates (Maurer *et al* 2006). High pH, high temperature, and long storage periods are required to produce safe and hygienic liquid fertilizer (Magri *et al* 2015; Hu *et al* 2016). Therefore, guidelines from the World Health Organization (WHO 2006) recommend a storage period of 6 months at 20 "Corhigher for the safe application of human urine on unrestricted crops. However, nitrogen loss via ammonia volatilization as a result of urea hydrolysis is another issue that should be addressed for the collection and transport of stored urine (Udert *et al* 2006). Ammonia volatilization not only decreases the efficiency of nitrogen recovery but also adversely affects environmental and human health (Galloway & Cowling 2002). In addition, the storage time of 6 months requires a large volume of storage tanks and is not cost-effective.

Hence, it is necessary to develop an efficient method that minimizes the required volume of storage tanks for human urine storage, as well as promoting the disinfection and stability of stored human urine. Although freezing concentrates almost 80% of the nutrients in urine to 25% of the original volume (Lind *et al* 2001), the utilization of frozen urine is problematic. Acidification or chemical oxidation (Hellström *et al* 1999; Zhang *et al* 2013) can inhibit urea decomposition; however, adding reagents to the urine is not an ideal solution. Temperature considerably affects urine storage and benefits pathogen elimination. For example, increasing storage temperature from 24 °Cto34 °C considerably decreases the number of enteric pathogens in diluted urine (Höglund *et al* 1998; Vin-neräs *et al* 2008). In addition, bacteria, such as *Salmonella typhimurium, Streptococcus faecalis,and Escherichia coli,*are inactivated at 65 °C within4 min (Fjendbo *et al* 1998). Mean-while, *Enterococcus faecalis* and *E. coli*are eliminated within 20 s under 65 °C (Spinks *et al* 2006). Moreover, solar pasteuri-zation at 55 °C decreases *E. coli* counts below the detectable limit (Dobrowsky *et al* 2015). Thus, utilizing the thermal effect on pathogen inactivation is an interesting alternative for human urine storage. Similar works on the disinfection of gray water, rainwater, and secondary effluent disinfection by solar energy showed that thermal disinfection is efficient (Amin *et al* 2014; Giannakis *et al* 2014; Lee *et al* 2016). Temperatures above 50 "Csignificantly affect the chemical hydrolysis of urea (Frankenberger & Tabatabai 1982). Urea hydrolysis is catalyzed by the urease produced by urease-producing bacteria (UPB); theoptimal temperatureof ureaseactivityisnormally 650C(Hagenkamp-Korth *et al* 2015). High temperatures, however, can reduce urea hydrolysis by inhibiting UPB growth. Therefore, the thermal storage of urine has potentially high sanitizing efficiency while minimizing urea hydrolysis.

Given that these advantageous properties have not been discussed in the presented literature, this study aims to investigate the thermal storage of human urine at 60 °C and 70 °C. It also aims to determine the effects of storage temperature on pathogen inactivation and urea decomposition. *E. coli* and fecal coliforms were used as the indicators of bacterial inactivation, whereas pH and ammonia were used as the main indicators of urea hydrolysis.

## MATERIAL AND METHODS

### Experimental set-up

**Figure 1:**
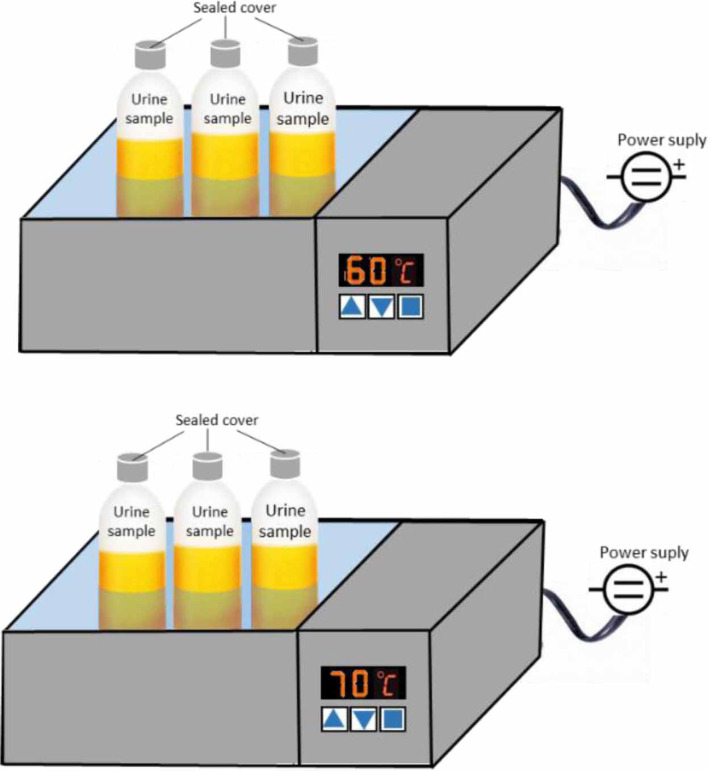
Experimental installation with water bath to maintain the temperature of urine storage.

Fresh urine samples were stored in 30 sealed 50-mL glass bot-tles to avoid cross-contamination during sampling. All the bottles were disinfected and dried prior to filling. The storage temperatures of 600C and 70 °C were controlled with a water bath. The urine samples were then cooled down to ambient temperature after a reasonable storage time. Urine that was stored at ambient temperature was used as control. The actual ambient temperature was measured daily. Each experimental scenario was maintained for several days until the end of the experiment. The physical, chemical, and biological parameters of the urine samples were tested. Figure 1 presents the installation set-up of the experiment.

### Urine samples

Fresh urine was collected from toilets at the School of Civil and Resource Engineering, University of Science and Technology, Beijing, China. To simulate a low-flushing urinal, the collected urine samples were diluted at a urine: distilled water ratio of 2:1 prior to distribution into experimental bottles. Given that urine was randomly collected in a plastic bucket from urinals with-out flushing water, the initial composition of fresh human urine varied from time to time. In this research, three experimental scenarios were investigated; thus, the initial characteristics of urine differed, as presented in Table 1.

**Table 1:** Main characteristics of fresh urine collected for the experimental scenarios

Scenarios	pH	Ammonia/ammonium (mg/L)	Fecal coliforms (CFU/L)	*E. coli* (CFU/L)
1	7.15	292.49	1.4 x 10^6^	2.0 x 10^4^
2	6.84	501.52	2.4 x 10^3^	2.0x 10^3^
3	7.26	331.43	3.4 x 10^4^	-

' means not detected.

### Sample analysis

#### Chemical analysis

pH was measured with a hand-held pH meter (HACH HQ30d, USA) and a corresponding electrode (pHC10101). Urine in glass bottles was measured immediately after sampling. Ammonia/ammonium content was analyzed calorimetrically with a DR/600 spectrophotometer (HACH, Co., USA) at 420 nm. All assays were performed in triplicate. Results were presented as averaged values.

#### Microbial analysis

There is currently no consensus on which organism(s) is the most useful hygiene indicator. Furthermore, there is no national regulation that mandates a single standard for urine reuse. According to the WHO Guidelines for the Safe Use of Wastewater, Excreta, and Greywater (WHO 2006), one or more of the total coliform, fecal coliform, and E. coli bacteria are used as wastewater effluent or reuse standards. Fecal coliforms originate from feces and are indicators of possible disease transmission (Fuhrmeister *et al* 2015); thus, fecal coliforms are often used as primary bacterial indicators. Moreover, the United States Environmental Protection Agency recommended *E. coli* and *Enterococci* as health risk indicators for water. Thus, fecal coliforms and *E. coli* were used as the core indicators in this study.

Initially, fecal coliform and *E. coli* in the urine samples were analyzed daily at the same sampling time. The sampling interval was adjusted during the later periods of storage. All the samples were diluted to a suitable concentration for parameter measurement.

The standard membrane filter method was used to quantify fecal coliform and E. coli (APHA 2012). An appropriate volume of urine sample was filtered through a 0.45-|im acetate cellulose filter. The filtered sample was placed in MFC broth and incubated at (44.5 ± 0.2) °C for 24 h. Blue colonies were counted to estimate the population of fecal coliforms. The number of fecal coliforms was presented as colony-forming unit per liter (CFU/L). To quantify *E. coli,* the filtered membrane was placed on Fuchsin basic sodium sulfite agar at 37 °C for 24 h and then transferred to NA-MUG agar for further incubation at 36 ± 1 °C for 4 h. Then, the bacteria were counted under a 366-nm ultraviolet lamp. Bacteria with blue fluorescence were counted to estimate the population of *E. coli.*

## RESULTS AND DISCUSSION

### Inactivation of bacteria

**Figure 2:**
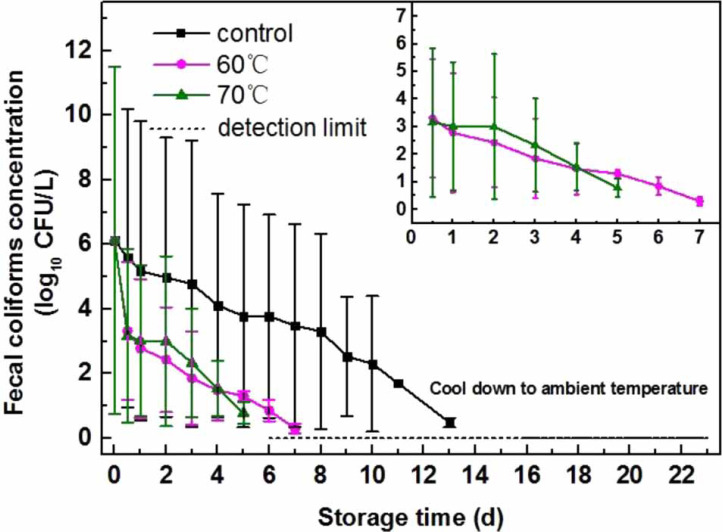
Fecal coliform concentration in the three urine samples during storage.

**Figure 3:**
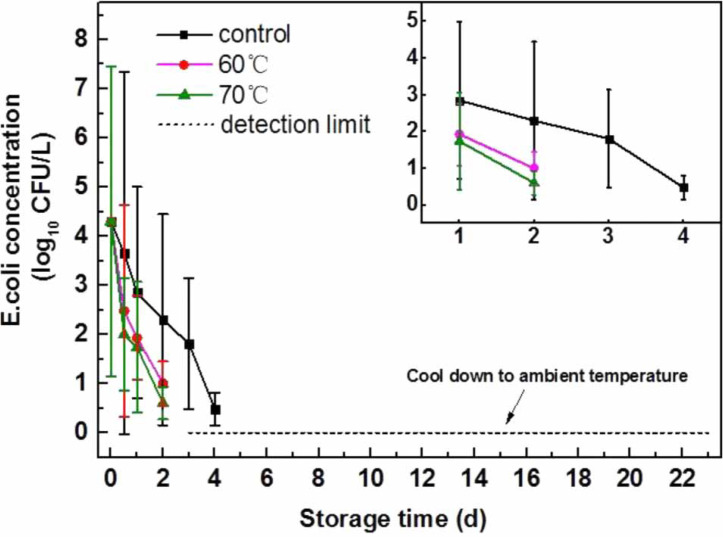
E. coli concentrations in the three urine samples during storage.

The overall storage time-dependence of the fecal coliform and E. coli concentrations in the three stored diluted urine samples is presented in Figures 2 and 3, respectively. An inset that represents the time-dependent elimination process is shown on the upper right quarter of the figures. Both fecal coKforms and E. coli decreased rapidly within the first week in urine that was stored at 60 °Cor70 °C compared with urine that was stored at ambient temperature.

Figure 2 demonstrates that the inactivation of fecal coli-forms in urine that was stored at ambient temperature was considerably slower than that in urine that was stored at high temperatures. In the former, the concentration of fecal coliforms decreased to undetectable levels on the 16th day of storage. The population of fecal coliforms in the urine samples that were stored at 60 °C and 70 °C decreased rapidly from 6 log to approximately 3 log on the 1st day of storage. The rate of population decline gradually stabilized and fecal coliform concentrations decreased to undetectable levels on the 8th and 6th days of storage. Similar results were obtained for E. coli. As shown in Figure 3, E. coli reached undetectable levels on the 3rd day of storage at 60 °C and 70 °C. By contrast, E. coli reached undetectable levels on the 5th day of storage at ambient temperature.

**Figure 4:**
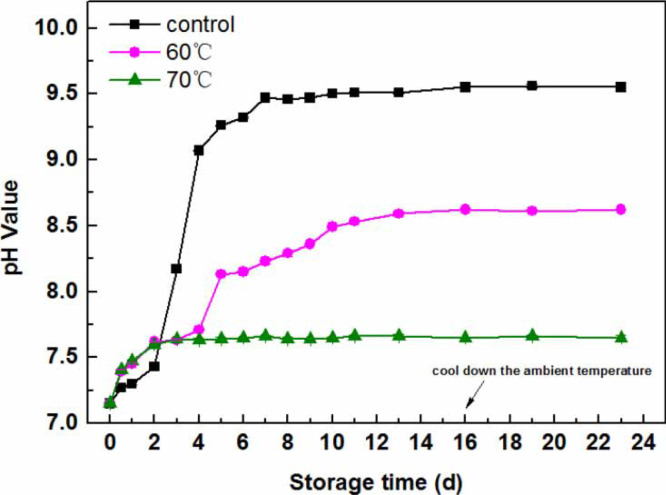
pH value of the diluted urine during the storage.

**Figure 5:**
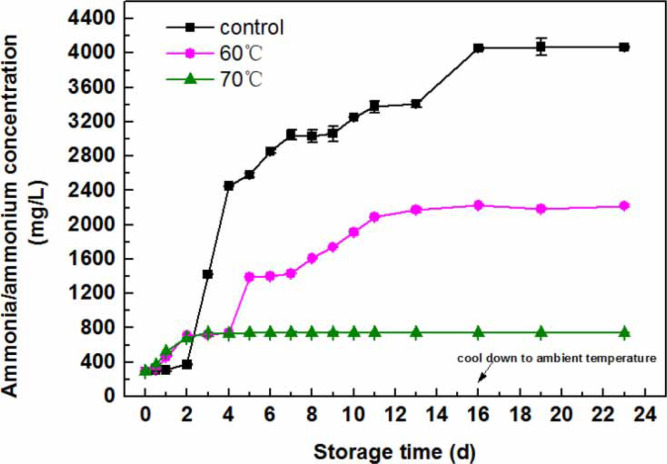
Ammonia/ammonium concentration of the diluted urine during storage.

The thermally stored urine was cooled down to ambient temperature. Then, the microbial indicators were continuously tested to evaluate the long-lasting disinfecting effect of high temperature. The three bacterial indicators were not reactivated in the two urine samples, which indi-cated that thermal storage has a stable disinfection effect. The experimental results showed that compared with the ambient temperature, high temperatures accelerate disinfection.

## Urea hydrolysis

To reflect urea hydrolysis, the storage time-dependence of pH values and the ammonia/ammonium concentrations of the three urine samples were investigated. The results are presented in Figures 4 and 5. The actual ambient temperature during the experimental period varied from 15 °C to 20.5 °C. The urine stored under ambient temperature had high pH (9.07) and high ammonia/ammonium concentration (2,453.32 mg/L) from the 4th day of storage onwards. Although both storage temperatures of 60 °C and 70 °C quickly removed fecal coliforms and *E. coli,* pH levels and ammonia/ammonium concentration differed at these temperatures. The pH value of the urine that was stored at 60 °C was initially 7.15, which then increased to 8.13 on the 5th day of storage, reached approximately 8.53 after 13 days of storage, and finally stabilized. Meanwhile, ammonia concentration increased from 292.49 mg/L to 1,392.59 mg/L before reaching approximately 2,173.04 mg/L. However, only a slight increase in pH and ammonia concentration was noted in the urine stored at 70 °C. pH increased from 7.15 to 7.60 and the ammonia concentration increased from 292.48 mg/L to 685.01 mg/L on the 2nd day of storage before stabilizing.

**Figure 6:**
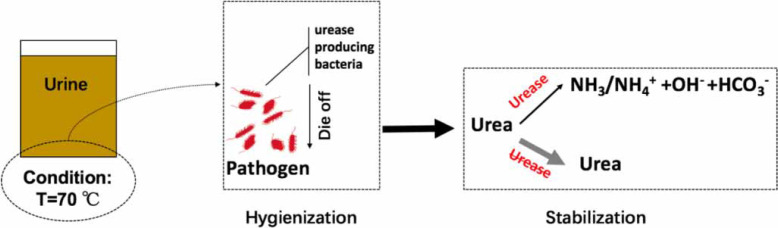
Process for disinfection and stabilization of human urine under thermal storage of 70 WC.

Under ambient storage conditions, the main process for disinfection is urea hydrolysis and high pH together with high ammonia/ammonium concentration, which are lethal to fecal coliforms and E. coli. In this experiment, the variation in pH and ammonia/ammonium concentration was correlated with the removal of fecal coliforms and E. coli. The thermal effect is responsible for pathogen inactivation in the thermally stored urine. Moreover, the high storage temperature caused the rapid removal of fecal coliforms and E. coli (Figures 2 and 3). Thermal storage has been hypothesized not only to enhance disinfection efficiency but also to prevent urea from hydrolyzing into ammonia/ammonium. Urea hydrolysis is catalyzed by urease from UPB. The thermal effect, how-ever, inactivates UPB, thus inhibiting urease production during storage. In this study, the inactivation of bacteria was slower at 60 °C than at 70 °C; thus, the remaining bacterial concentration in the urine that was stored at 60 °C was higher than that of the urine that was stored at 70 °C. As a result, more UPB remained in the urine that was stored at 60 °C during early storage. Therefore, urea hydrolysis occurred. Although temperatures of both 70 °C and 60 °Cinfluenced urease activity, 70 °C had a more significant effect on urease because the optimal temperature for urease activity is 65 °C(Hagenkamp-Korth *et al* 2015) and UPB is less active. The reduction of urease concentration via UPB inactivation likely contributes more to the inhibition of urea hydrolysis. Therefore, considering disinfection effect and urea hydrolysis, storage at 70 °C for 7 days produces the optimal pathogenic bacteria inacti-vation and urea stabilization. The storage process is as described in Figure 6.

### Replication tests

**Figure 7:**
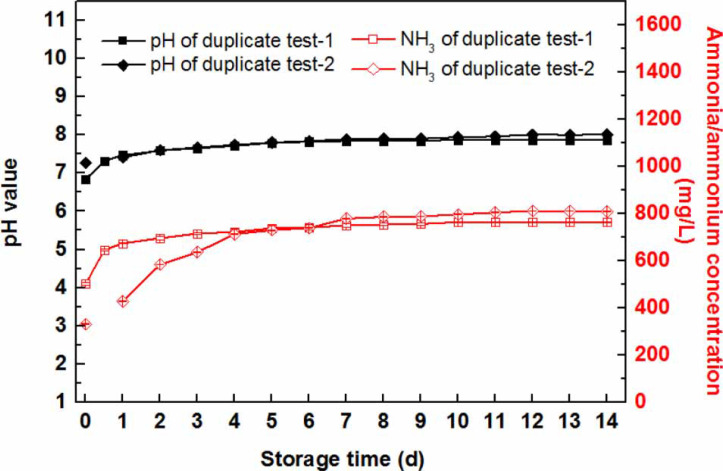
pH values and ammonia/ammonium concentrations in the two urine samples.

Based on the above experimental results, thermal storage, i.e., storing urine at 70 °C for 7 days, was applied to human urine in two duplicate tests to test the reproduci-bility of the selected operational conditions. Heated urine was naturally cooled down to ambient temperature for another 7 days to investigate the disinfection and stabilization effect of thermal storage. The experimental results for the thermal storage of the two urine samples are presented in Figure 7.

Fecal coliforms and *E. coli* in the two urine samples were completely inactivated within 2 days of storage. Fecal coliforms and *E. coli* decreased to undetectable levels after the first storage day, particularly in duplicate-1. For urea hydrolysis, little variation was found in the pH and ammo-nia/ammonium concentration of the two urine samples during the 7 days of storage. These results further supported the efficiency and reproducibility of the previous experimental results. The pH values of the two urine samples on the 7th day of storage were 7.84 and 7.89, whereas the ammonia concentrations were 749.39 and 778.46 mg/L, i.e., only a slight increase was observed. Moreover, disinfection and stability were retained even after storing the urine under ambient temperature, as evidenced by the lack of significant changes in the microbial, physical, and chemical character-istics of the samples.

Thermal treatment is a potential alternative technology for community toilets with source-separated systems. In fecal sludge composting, applying high temperatures for a short duration may be as effective as applying low temperatures for a longer duration (Day *et al* 2001). Given that similar operational parameters are suitable for urine storage, increasing temperature from the ambient to 70 °C during the thermal storage of urine can minimize storage tank volume. Thus, the thermal storage of urine is suitable for commu-nities with limited space and is a potential, inexpensive solution for some solar energy-rich regions. Moreover, this system can be heated with renewable energy, such as biogas and ground source heat. The present study proved that the thermal treatment of human urine at 70 °C for 7 days for disinfection and stabilization can be achieved tech-nically. However, a more comprehensive study on the tradeoff between energy costs, environmental impact/ resource use from heating with traditional energy sources (compared with renewable energies), and efficient nutrient recovery from a waste stream should be discussed before the practical application of thermal storage for human urine.

## CONCLUSIONS

In this study, the thermal treatment of urine at 60°C and 70°C was conducted and evaluated in terms of disinfection and stabilization. Storage at 70 °C for 7 days realized optimal pathogenic bacteria inactivation and urea stabilization. High storage temperature had multiple advantages for human urine treatment, including: (1) acceleration of pathogen inactivation; (2) inhibition of urea decomposition; and (3) minimization of storage tank volume. Urea hydrolysis was likely inhibited by the decreased UPB count because urease production became limited.

Further research should be conducted before this storage system can be used for practical applications. Future studies should: (1) use more indicators for sanitation monitoring to broaden the spectrum of cross-sectional pathogenic inacti-vation efficiency to find an optimal storage temperature; (2) investigate the mechanism of microorganism inactivation during high-temperature storage; (3) compare the loss of different gaseous nitrogen forms from thermal storage with that from conventional storage; and (4) analyze energy balance and minimize energy consumption.
